# Objective vestibular function changes in children following cochlear implantation

**DOI:** 10.3233/VES-190763

**Published:** 2022-01-11

**Authors:** Ruijie Wang, Xiuhua Chao, Jianfen Luo, Daogong Zhang, Jiliang Xu, Xianfeng Liu, Zhaomin Fan, Haibo Wang, Lei Xu

**Affiliations:** a Department of Otolaryngology-Head and Neck Surgery, Shandong Provincial ENT Hospital, Cheeloo College of Medicine, Shandong University, Jinan, P.R. China; b Department of Auditory Implantation, Shandong Provincial ENT Hospital, Jinan, P.R. China

**Keywords:** Cochlear implantation, children, LVAS, vestibular function

## Abstract

**BACKGROUND:**

To date, systematically objective evaluations of vestibular function in children with cochlear implantation (CI) have been conducted sparsely, especially in children with large vestibular aqueduct syndrome (LVAS).

**OBJECTIVE:**

Our goal was to investigate the function of all five vestibular end-organs pre- and post-cochlear implantation in children with LVAS and normal CT.

**METHODS:**

In this retrospective cohort study, 34 children (age 4–17 years) with bilateral profound sensorineural hearing loss (SNHL) undergoing unilateral CI were included. Participants included 18 (52.9%) children with LVAS. Objective modalities to evaluate vestibular function included the caloric test, cervical vestibular-evoked myogenic potentials (cVEMP), ocular vestibular-evoked myogenic potentials (oVEMP), and video head impulse test (vHIT). All measurements were performed before surgery and 9 months after surgery.

**RESULTS:**

Mean age at CI was 8.1±3.7 years. Caloric testing showed hypofunction in 38.2% of cases before implantation and in 50% after (*p* > *0.05*). We found a significant increase of overall abnormality rate in cVEMP and oVEMP from pre- to post-CI (*p* < *0.05*). In all three semicircular canals tested by vHIT, there were no statistically significant mean gain changes (*p* > *0.05*). Higher deterioration rates in cVEMP (53.3%) and oVEMP (52.0%) after surgery were observed (*p* < *0.05*). In children with LVAS, cVEMP revealed a higher deterioration rate than superior semicircular canal (SSC) and posterior semicircular canal (PSC) (*p* < *0.05*). In children with normal CT, the deterioration rates in VEMPs were both higher than those in vHIT (*p* < *0.05*).

**CONCLUSIONS:**

In general, the otolith organs were the most affected peripheral vestibular sensors in children after cochlear implantation. The variations in otolith function influenced by CI were different between children with LVAS and normal CT. We recommend the use of this vestibular function test battery for children with cochlear implantation.

## 1 Introduction

Cochlear implantation (CI), as a surgically im-planted electronic device, restores hearing ability of patients with bilateral severe to profound sensorineural hearing loss (SNHL). Although CI is a safe surgical procedure, postoperative vestibular dysfunction may occur and the main reason is still unknown. It has been reported that CI surgery and electrical activity are associated with the effect on vestibular sensors due to the close anatomic and physiologic relationships of the cochlea and vestibular system [12]. Possible mechanisms leading to vestibular dysfunction may include: 1) Direct trauma caused by electrode insertion; 2) Acute serous labyrinthitis due to cochleostomy; 3) Intraoperative loss of perilymph; 4) Foreign body reaction with labyrinthitis; 5) Endolymphatic hydrops; 6) Electrical stimulation from the implant itself [4, 17, 27, 39, 47].

Patients with large vestibular aqueduct syndrome (LVAS) usually have sudden, fluctuating, or progressive hearing loss since childhood [35, 49]. With the development of minimally invasive surgery, more and more children with LVAS may need cochlear implantation after hearing loss. It has been reported that vestibular dysfunction is common in patients with LVAS, but only 45% of them have signs and symptoms [55]. However, it is unknown whether vestibular function in children with LVAS is affected by CI. Therefore, it is necessary to use objective methods to assess it.

Balance relies on the integration of sensory inputs from the vestibular, visual and somatosensory systems [36]. The vestibulo-ocular system is responsible for gaze stabilization during head movements. The vestibulospinal system contributes to muscle tone, which is necessary for the emergence of early motor function, as well as aiding postural control [41]. Vestibular dysfunction affects balance and perception abilities, including gross motor function, visuospatial ability, memory, attention, and executive function [7, 22, 26]. Many studies have documented that children with SNHL are more likely to have associated peripheral vestibular dysfunction [13, 31]. With the increase of unilateral or bilateral cochlear implantation in children with SNHL, the risk of vestibular dysfunction needs to be carefully taken into account. To date, debates surrounding the effect of CI on the vestibular system in children with SNHL still continue [54].

Recently, in studies of vestibular function in children with CI, horizontal semicircular canal (HSC) function has been evaluated by caloric testing and saccular function has been tested by cervical vestibular-evoked myogenic potentials (cVEMP) [2, 14, 20, 23, 24, 30, 47, 53, 54]. However, there are few reports on utricular function [29, 53]. The video head impulse test (vHIT) is a fast, practical, and noninvasive test that can be used to evaluate all three semicircular canals [52]. Although studies have demonstrated that all three semicircular canals can be tested by HIT [2, 25], there is still a lack of available data concerning vHIT [37]. In order to acquire a more comprehensive understanding of vestibular function, all five peripheral vestibular end organs must be considered simultaneously.

At present, systematic studies on changes in vestibular function in children after CI are few, and the alterations of vestibular function in children with LVAS have been seldomly reported. Therefore, this research systematically investigated the potential effect of CI on all five vestibular end-organs in children, especially in children with LVAS.

## 2 Materials and methods

### 2.1 Subjects

This study is a retrospective study of 34 children (34 ears) who underwent unilateral CI in the Auditory Implantation Department of Shandong Provincial ENT Hospital Affiliated to Shandong University from November 2015 to November 2018. Across all subjects, the mean age at implantation was 8.1±3.7 years (range: 4–17 years). The indication for CI was based on severe-to-profound bilateral deafness with no significant improvement from hearing aids. Patients were excluded if they were ≥18 years, unable to participate in the vestibular assessments or had undergone previous otologic surgery. Computerized tomography (CT) scans and magnetic resonance imaging (MRI) of the temporal bones were performed before surgery. The surgical technique was performed by one senior surgeon. The surgical approaches were chosen according to the electrode types. Patients underwent CI via two surgical approaches: the round window (RW) and extended RW approach. The RW approach was used for the Nucleus 422, Nucleus CI24REST, Med-EL FLEX 28, and Nurotron CS-10A electrode arrays. The extended RW approach was used for the Nucleus CI24RECA electrode array. All children received minimally invasive unilateral implantation.

Vestibular function of the implanted ears was evaluated before CI and 9 months after CI. During the tests, CIs were all switched off after processor activation. The objective methods for evaluating vestibular function were as follows.

### 2.2 Caloric test

The bithermal caloric test was performed. A video-based system was used (Ulmer VNG, v. 1.4; SYNAPSYS, Marseille, France) to acquire and analyze the eye response. Each ear was irrigated alternatively with a constant flow of air at 24°C and 49°C for 40 seconds. The response was recorded over 3 minutes. A 7-minute interval between each stimulus was used to avoid cumulative effects. The maximum slow-phase velocity (SPV) of nystagmus after each irrigation was calculated. Unilateral weakness (UW) was determined according to Jongkee’s formula. In our laboratory, a value of UW less than 20% was considered to be normal. The value of UW more than 90% was considered to be canal paresis.

### 2.3 cVEMP test

cVEMPs were recorded using Neuro-Audio auditory evoked potential equipment (Neurosoft LTD, Ivanov, Russia). The test was performed with the patient seated. Tone burst stimuli (95 dB nHL, 500 Hz) were delivered via a standard insert earphone (EAR-3A). Active recording electrodes with respect to the examination were placed on the region of the upper third of the sternocleidomastoid muscle (SCM) on both sides. The reference electrodes were placed on the upper sternum. The ground electrode was on the nasion. The head was rotated toward the contralateral side of the stimulated ear to achieve tonic contraction of the SCM during recording. The stimulation rate was 5.1 Hz. Bandpass filtering was 30–2000 Hz. An amplitude ratio over 30% was considered abnormal if the weaker response was from the implanted ear. In the implanted ear, absent responses were considered abnormal.

### 2.4 oVEMP test

Ocular vestibular-evoked myogenic potentials (oVEMPs) were recorded using Neuro-Audio auditory evoked potential equipment (Neurosoft LTD, Ivanov, Russia). The electromyographic activity of the extraocular muscle was recorded with the patient in the seated position. Tone burst stimuli (95 dB nHL, 500 Hz) were delivered via a standard insert earphone (EAR-3A). The active recording electrodes were placed on the infra-orbital ridge 1 cm below the center of each lower eyelid. The reference electrodes were positioned approximately 1 cm below them. The ground electrode was on the nasion. The results were recorded with eyes open and maximal gaze upward. The stimulation rate was 5.1 Hz. Bandpass filtering was 1–1000 Hz. An amplitude ratio over 30% was considered abnormal if the weaker response was from the implanted ear. In the implanted ear, absent responses were considered abnormal.

### 2.5 vHIT test

The vHIT device (Ulmer II Evolution, France) was used. The VHIT Ulmer II was equipped with an ultra-sensitive camera that filmed the patient’s face from a distance of approximately 90 cm. The patient was instructed to maintain eye focusing on a stationary object on a screen at about 1 m distance while examiner manipulated the patient’s head with quick, and precise head movements. The vestibulo-ocular reflex (VOR) gain was calculated by vHIT software based on head velocity and eye velocity curves. When the head was turned to one side in the plane of semicircular canal to be tested, the VOR maintained visual fixation. The breaking of visual fixation, shown by a corrective saccade, indicated a lateral canal disorder. This test was possible as soon as the child could hold their head steady. In a full test, 5–10 head thrusts were completed per canal for the recording. The VOR gain of HSC less than 0.8 was considered to be abnormal. VOR gains of the superior semicircular canals (SSC) and posterior semicircular canal (PSC) less than 0.7 were considered to be abnormal.

### 2.6 Statistical analyses

Statistical analysis of the data was performed using the Statistical Package for the Social Sciences (SPSS), version 17.0 (SPSS, Inc., Chicago, IL). Variables of the overall results were compared using the McNemar test. The Chi-square test was used to compare the deterioration rates. Statistical significance was considered as *p* < *0.05*.

## 3 Results

Thirty four children who underwent CI (22 males and 12 females; age range, 4 to 17 years; mean 8.1±3.7 years) participated in this study, including 18 participants with LVAS (4 to 15 years; mean 7.2±3.1 years), and 16 participants with normal CT findings (5 to 17 years; mean 9.3±4.2 years). The demographic characteristics of all participants are described in Table 1 Each of implanted electrodes achieved full insertion without any resistance or complication. The overall evaluation results before and 9 months after CI are described in Table 2.

**Table 1:** Demographic characteristics of the 34 patients in this study

Mean age at implantation	8.1±3.7 (range 4–17)
Gender
Male	22 (64.7%)
Female	12 (35.3%)
Hearing loss origin
Congenital	26 (76.5%)
Progressive	8 (23.5%)
Implant side
Left	15 (44.1%)
Right	19 (55.9%)
Surgical approach
RW	18 (52.9%)
Extended RW	16 (47.1%)
CT scan
Normal	16 (47.1%)
EVA	18 (52.9%)
Implant
Nucleus CI24RECA	16 (47.1%)
Nucleus CI24REST	3 (8.8%)
Nucleus 422	5 (14.7%)
Med-EL FLEX28	4 (11.8%)
Nurotron CS-10A	6 (17.6%)

RW: round window, EVA: enlargement of the vestibular aqueduct.

**Table 2:** The normal objective evaluation results of vestibular function pre-CI and 9 months post CI (implanted side, patients, n%)

	Pre-CI (n, %)			9 months post CI (n, %)		
Test	All	LVAS	Normal CT	All	LVAS	Normal CT
Caloric	21 (61.8)	11 (61.1)	10 (62.5)	17 (50.0)	6 (33.3)	11 (68.8)
cVEMP	30 (88.2)	14 (77.8)	16 (100.0)	16 (47.1)	9 (50.0)	7 (43.8)
oVEMP	25 (73.5)	13 (72.2)	12 (75.0)	14 (41.2)	10 (55.6)	4 (25.0)
vHIT
HSC	32 (94.1)	17 (94.4)	15 (93.8)	31 (91.2)	15 (83.3)	16 (100.0)
SSC	34 (100.0)	18 (100.0)	16 (100.0)	33 (97.1)	17 (94.4)	16 (100.0)
PSC	33 (97.1)	17 (94.4)	16 (100.0)	32 (94.1)	16 (88.9)	16 (100.0)

n: number; cVEMP: cervical vestibular-evoked myogenic potential, oVEMP: ocular vestibular-evoked myogenic potential, vHIT: video head impulse test; HSC: horizontal semicircular canal, SSC: superior semicircular canal, PSC: posterior semicircular canal; All: all the 34 children, LVAS: children with LVAS (large vestibular aqueduct syndrome), Normal CT: children with normal CT scan.

### 3.1 The overall abnormality rates through all the four measurements

**Fig. 1:**
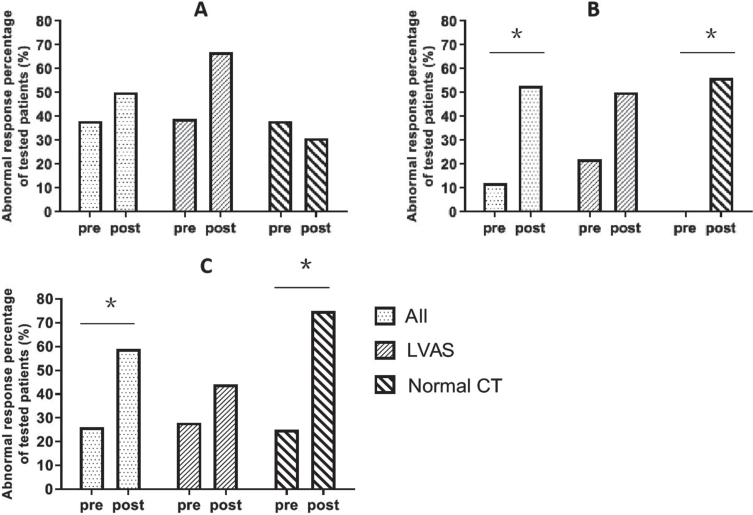
The variations of vestibular function test results (implanted side, patients, n%). (A) Caloric test. (B) cVEMP. (C) oVEMP. All: all the 34 children, LVAS: children with LVAS, Normal CT: children with normal CT. Mc Nemar test: Pre vs post, Y axis: abnormal response percentage of tested patients (%), pre: pre-CI, post: 9 months post CI, ^*^p < *0.01*.

Of all the 34 children, 13 (38.2%) showed abnormal results before CI and 17 (50.0%) showed abnormal results at 9 months postoperatively for the caloric test (*p* = *0.388*); for cVEMP, four (11.8%) patients showed dysfunction before CI and the numbers increased to 18 (52.9%) after CI (*p* = *0.001*); for oVEMP, nine patients (26.5%) showed dysfunction before CI and the numbers increased to 20 (58.8%) after CI (*p* = *0.007*) (Fig. 1). In all three semicircular canals tested by vHIT, there was no statistical significance of mean gain change at 9 months postoperatively (*p* > *0.05*).

Of the 18 children with LVAS, seven (38.9%) showed abnormal results on caloric testing before surgery and 12 (66.7%) showed abnormal results after surgery (*p* = *0.125*); four children (22.2%) showed abnormal results in cVEMP before surgery and nine children (50.0%) showed abnormal after surgery (*p* = *0.180*); five children (27.8%) showed abnormal results in oVEMP before surgery and eight (44.4%) showed abnormal after surgery (*p* = *0.453*) (Fig. 1). In all three semicircular canals tested by vHIT, there was no statistical significance of mean gain change at 9 months postoperatively (*p* > *0.05*).

Of the 16 children with normal CT findings, the caloric test results of 6 children (37.5%) were abnormal before implantation and those of 5 (31.3%) were abnormal after implantation (*p* = *1.000*); the cVEMP results of all 16 children (100.0%) were normal before implantation but those of 9 (56.3%) were abnormal after implantation (*p* = *0.004*); the oVEMP results of 4 children (25.0%) were abnormal before implantation but those of 12 (75.0%) were abnormal after implantation (*p* = *0.008*) (Fig. 1). In all three semicircular canals tested by vHIT, there was no statistical significance of mean gain change at 9 months postoperatively (*p* > *0.05*).

It could be concluded that the abnormality rate (38.2%) of the caloric test was high before surgery. The results of caloric testing at 9 months postoperatively were as follows: (1) Among the 13 patients with abnormal responses on caloric testing preoperatively, nine showed no change, and the remaining four turned from abnormal responses to normal responses. (2) Among the 17 patients with postoperatively abnormal results on caloric testing, nine were from the original 13 patients.

### 3.2 The deterioration rates of all the five vestibular end-organ functions at 9 months postoperatively

**Fig. 2:**
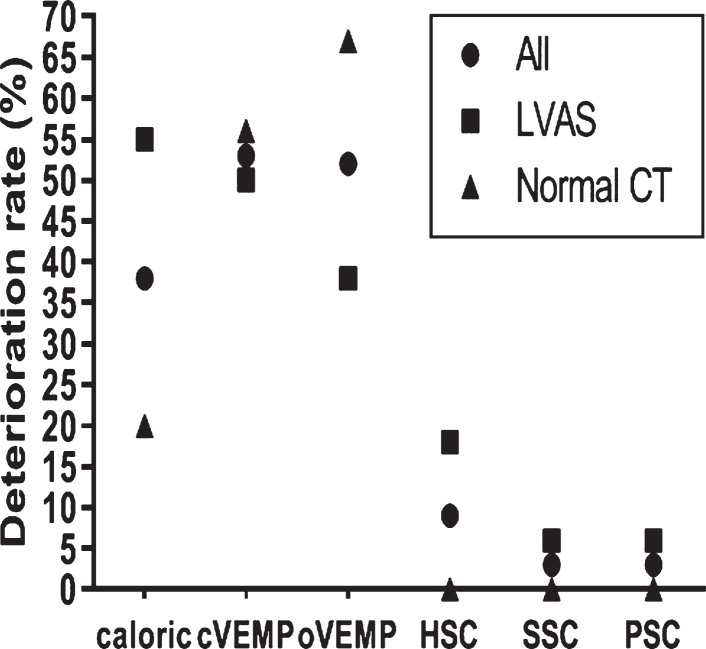
The deterioration rates of all the five vestibular end-organ functions at postoperative month 9 (%). Chi-square test, All (caloric test vs SSC and PSC, *p* < *0.05*; cVEMP vs HSC, SSC and PSC, *p* < *0.05*; oVEMP vs HSC, SSC and PSC, *p* < *0.05*), LVAS (caloric test vs SSC and PSC, *p* < *0.05*; cVEMP vs SSC and PSC, *p* < *0.05*), Normal CT (cVEMP vs HSC, SSC and PSC, *p* < *0.05*; oVEMP vs HSC, SSC and PSC, *p* < *0.05*).

Among all the 34 children, 21 children showed normal results on the caloric test before CI, eight were found to have vestibular dysfunction after CI, and the deterioration rate was 38.1%. The deterioration rate was 53.3% for cVEMP, 52.0% for oVEMP, 9.4% for HSC (vHIT), 2.9% for SSC, and 3.0% for PSC. HSC tested by the caloric test showed a higher deterioration rate than SSC and PSC tested by vHIT (*p* < *0.05*). VEMPs showed higher deterioration rates than vHIT (*p* < *0.05*) (Fig. 2).

In the 18 children with LVAS, the deterioration rate was 54.5% for caloric testing, 50.0% for cVEMPs, 38.5% for oVEMPs, 17.6% for HSC (vHIT), 5.9% for SSC, and 5.9% for PSC. The deterioration rate was higher for caloric testing than those for SSC and PSC (*p* < *0.05*), and the deterioration rate in cVEMP was higher than those for SSC and PSC (*p* < *0.05*) (Fig. 2).

In the 16 children with normal CT findings, the deterioration rate was 20.0% for caloric testing, 56.3% for cVEMP, 66.7% for oVEMP, 0.0% for HSC (vHIT), 0.0% for SSC, and 0.0% for PSC. VEMPs showed higher deterioration rates than those of vHIT (*p* < *0.05*).

## 4 Discussion

CI has become a routine procedure for children with bilateral severe to profound sensorineural hearing loss [28]. It is known that CI may potentially lead to saccular dysfunction in pediatric patients [54]. However, the data regarding the effect of CI on utricular function in children is limited [29, 53]. The vHIT, a new test, not only evaluates HSC but also the other two semicircular canals. There is no single objective test that is sensitive enough to be used as a unique test [1]. In order to evaluate overall vestibular function, the caloric test, cVEMP, oVEMP, and vHIT were used in this study.

In this research, the overall abnormality rates before and after CI as well as deterioration rates of all five end-organs were analyzed. The postoperative abnormality rate was 50% for caloric testing, 52.9% for cVEMPs, and 58.8% for oVEMPs. The deterioration rate was 38.1% for caloric testing, 53.3% for cVEMPs, and 52.0% for oVEMPs after CI. Previous research has revealed that most pediatric subjects had reduced responses of caloric testing and VEMPs after implantation; these findings were similar to ours [14, 23, 24, 29, 30, 53, 54]. A study demonstrated that the rate of caloric dysfunction was 16.66% after CI [20]. A higher deterioration percentage (38.1%) was found in ours. It was reported that the postoperative response rate was 19.2% for oVEMPs and 34.8% for cVEMPs [53]. In this study, the overall abnormality rate after CI was as high as 52.9% for cVEMPs and 58.8% for oVEMPs.

Changes in cochlea function during implantation could potentially cause changes in the semicircular canals and otolith organs since the auditory and vestibular organs share the same fluid [38]. In our series of studies, a high abnormality rate (38.2%) before surgery and no statistically significant variation after surgery were found for caloric testing, although the overall abnormality rate was from 38.2% to 50.0%. A significant increase of overall abnormality rate for VEMPs and no changes in all three semicircular canals tested by vHIT were discovered. Although there was no statistical difference in deterioration rate between caloric testing and VEMPs, there was a trend that the rates were higher in VEMPs (53.3% in cVEMP, 52.0% in oVEMP). Our result revealed that there were more impairments in both saccular and utricular function in children after CI, which is consistent with previously published results [29, 53]. It was speculated that the deterioration of otolith function after implantation was the most serious, especially the saccule. The saccule is more susceptible to damage than the utricule or semicircular canals because it is close to the insertion pathway of the electrode [48, 51]. This proximity may make the saccule more vulnerable to surgical damage in the presence of electrode insertion, drilling or variations in inner ear fluid environments. A previous study demonstrated that CI did not impair saccular and HSC functions in children aged 1–4 years old without activating the processors. The RW surgical approach was verified to have no disturbing effect on vestibular function in children [2]. Our result is inconsistent with this finding. Besides the limited participants in the previous study, we used both RW and extended RW surgical approaches. The RW technique has been considered safer than cochleostomy [43]. In addition to surgical factors, patients were monitored with the processor on for a longer period in this study. It is known that secondary and distant effects of CI may threaten vestibular function, such as by inflammation, fibrous tissue formation, or ossification [16]. Regarding the effect of electrical stimulation on vestibular function, it may impart pathologic changes to the inner ear or provide conflicting sensory inputs, leading to negative effects. However, some research showed that CI might have a positive potential to elicit VEMPs [39]. Recently, another paper revealed that the RW surgical approach had an adverse influence on otolith function in children without activating the processor [29]. Therefore, the possible mechanism of vestibular dysfunction influenced by CI remains unclear. Although vestibular dysfunction was not correlated with core symptoms [10], parents should still be advised to pay more attention to postoperative relative vertigo symptoms, balance problems, and functional rehabilitation of children.

In contrast to previous studies, we also evaluated vestibular function in children with LVAS and children with normal CT findings, separately. We found a significant increase of overall abnormality rate in VEMP from pre- to post-CI in children with normal CT, but no significant change in children with LVAS. VEMPs showed higher deterioration rates than vHIT in children with normal CT. For children with LVAS, only cVEMP revealed a higher deterioration rate than SSC and PSC. Our data suggested that the variations of otolith function influenced by CI were different between children with LVAS and children with normal CT. In children with LVAS, the pressure generated during the insertion of electrodes could be released through the enlarged vestibular aqueduct or released into the endolymph fluid, leading to less impairment. Besides the vestibular dysfunction, the peripheral mechanical changes should be considered. The insertion or presence of electrodes is known to alter the pressure of the middle ear system [18, 19, 45]. The occurrence of air-bone gaps (ABG) adverstly affects hearing preservation after CI [3, 11, 32, 33]. Even a small gap of 5 to 10 dB could abolish the air conduction stimuli (ACS) responses in patients with normal vestibular function [34]. This conductive impairment is related to the change in middle ear function, or cochlear mechanics [3, 40]. Researchers documented lower thresholds in cVEMP and higher amplitudes in oVEMP in patients with LVAS [46, 57]. VEMPs have also been reported to be present in the ears of patients with ABG and LVAS [44]. We hypothesized that CI affected ABG in a different manner in children with LVAS. It is important to note that these mechanical impacts of CI on the peripheral auditory system varied from patient to patient and from ear to ear [34]. Therefore, the mechanism leading to the different performances of otolith function is still unknown. Our findings may help clinicians gain a new understanding of children with LVAS.

In this work, we evaluated all three semicircular canals functions under high frequency impulse stimulation. Meanwhile, the HSC function was evaluated under low frequency stimulus. Although the caloric test demonstrated a high preoperative dysfunction rate, HSC tested with caloric showed a higher deterioration rate than SSC and PSC tested by vHIT. All three semicircular canals tested by vHIT showed no significant differences in the deterioration rates. The deterioration rate was higher in HSC tested by caloric (38.1%) than HSC tested by vHIT (9.4%), although there was no statistically significant difference. The reason for the contradictory results on HSC may be the difference in methods. The caloric and vHIT tests measure two extreme frequency ranges of the horizontal VOR. vHIT uses a physiological stimulus with higher testing frequencies (>1 Hz, caloric test < 0.003 Hz), close to the physiological stimuli in daily life, while the caloric test uses a non-physiological stimulus [56]. Zellhuber et al. concluded that there were different parallel recovery processes for vestibular function between the two tests [56]. Our results demonstrated that the impairment of HSC function could be tested under a non-physiological stimulus. In order to acquire an overall evaluation of semicircular canal functions, these two methods should be used together.

Different results among previous studies were attributed to the age of patients investigated, cause of deafness, surgical technique, type of device, evaluation timing, operator, or sensitivity of examination method between laboratories. The timing of evaluation varied from 2 days to 2 years [6, 8, 21]. It seemed that patients over 60 had more vestibular impairment after surgery [5, 9, 15]. Other factors such as the cause of deafness, surgical technique or the type of device seem to make no differences [5, 42]. In this report, children with LVAS and normal CT showed inconsistent alterations of otolith function. We hypothesized that the cause of deafness might affect vestibular function. Regarding the surgical approach, all participants underwent a minimally invasive surgical approach instead of cochleostomy. Available evidence suggests that minimally invasive surgery reduces the intraoperative damage, avoids the mixture of labyrinthine fluids, and preserves the functional integrity of the inner ear to a greater extent [50].

## 5 Limitations

The present study was a systematic research study of both canal and otolith function in children with CI, and LVAS children were considered at the same time. There were two reports on vestibular function in children with RW surgical approach and without activating the processor before [2, 29]. Our results are consistent with one study, whereas inconsistent with another. More than half of the patients were treated with RW approach in this study and all children were analyzed under a long use of processor. Further study should analyse the vestibular function in children following RW surgical approach with and without activating the processor.

## 6 Conclusion

In summary, CI led to different influences on peripheral vestibular function in children, and saccular and utricular function were seriously affec-ted. Surprisingly, the variations in otolith function influenced by CI were different between children with LVAS and normal CT. All the four objective tests should be used together as a gold standard vestibular battery.

## References

[1] Abouzayd M. , Smith P.F. , Moreau S. and Hitier M. , What vestibular tests to choose in symptomatic patients after a cochlear implant? A systematic review and meta-analysis, Eur Arch Otorhinolaryngol 274 (2017), 53–63. 27059840 10.1007/s00405-016-4007-4

[2] Ajalloueyan M. , Saeedi M. , Sadeghi M. and Zamiri F. , Abdollahi, The effects of cochlear implantation on vestibular function in 1–4 years old children, Int J Pediatr Otorhinolaryngol 94 (2017), 100–103. 28166997 10.1016/j.ijporl.2017.01.019

[3] Banakis Hartl R.M. , Mattingly J.K. , Greene N.T. , Jenkins H.A. , Cass S.P. and Tollin D.J. , A preliminary investigation of the air-bone gap: Changes in intracochlear sound pressure with air- and bone-conducted stimuli after cochlear implantation, Otol Neurotol 37 (2016), 1291–1299. 27579835 10.1097/MAO.0000000000001184 PMC5089803

[4] Bance M.L. , O’Driscoll M. , Giles E. and Ramsden R.T. , Vestibular stimulation by multichannel cochlear implants, Laryngoscope 108 (1998), 291–294. 9473085 10.1097/00005537-199802000-00025

[5] Basta D. , Todt I. , Goepel F. and Ernst A. , Loss of saccular function after cochlear implantation: the diagnostic impact of intracochlear electrically elicited vestibular evoked myogenic potentials, Audiol Nurootol 13 (2008), 187–192. 10.1159/000113509 18212494

[6] Batuecas-Caletrio A. , Klumpp M. , Santacruz-Ruiz S. , Gonzale F.B. , Sanchez G.E. and Arriaga M. , Vestibular function in cochlear implantation: Correlating objectiveness and subjectiveness, Laryngoscope 125 (2015), 2371–2375. 25891786 10.1002/lary.25299

[7] Bigelow R.T. and Agrawal Y. , Vestibular involvement in cognition: visuospatial ability, attention, executive function, and memory, J Vestib Res 25 (2015), 73–89. 26410672 10.3233/VES-150544

[8] Bonucci A.S. , Costa Filho O.A. , Figueiredo Mariotto L.D. , Bortoleto Amantini R.C. and Alvarenga Kde F. , Vestibular function in cochlear implant users, Braz J Otolaryngol 74 (2008), 273–278. 10.1016/S1808-8694(15)31100-9 PMC9442058 18568208

[9] Brey R.H. , Facer G.W. , Trine M.B. , Lynn S.G. , Peterson A.M. and Suman V.J. , Vestibular effects associated with implantation of a multiple channel cochlear prosthesis, Am J Otol 16 (1995), 424–430. 8588641

[10] Chen X. , Chen X.H. , Zhang F. and Qin Z.B. , Influence of cochlear implantation on vestibular function, Acta Otolaryngol 136 (2016), 655–659. 27008103 10.3109/00016489.2016.1154186

[11] Chole R.A. , Hullar T.E. and Potts L.G. , Conductive component after cochlear implantation in patients with residual hearing conservation, Am J Audiol 23 (2014), 359–364. 25165991 10.1044/2014_AJA-14-0018

[12] Cushing S.L. , Gordon K.A. , Rutka J.A. , James A.L. and Papsin B.C. , Vestibular end-organ dysfunction in children with sensorineural hearing loss and cochlear implants: an expanded cohort and etiologic assessment, Otol Neurotol 34 (2013), 422–428. 23370550 10.1097/MAO.0b013e31827b4ba0

[13] Cushing S.L. , Papsin B.C. , Rutka J.A. , James A.L. and Gordon K.A. , Evidence of vestibular and balance dysfunction in children with profound sensorineural hearing loss using cochlear implants, Laryngoscope 118 (2008), 1814–1823. 18758383 10.1097/MLG.0b013e31817fadfa

[14] Devroede B. , Pauwels I. , Le Bon S.D. , Monstrey J. and Mansbach A.L. , Interest of vestibular evaluation in sequentially implanted children: preliminary results, Eur Ann Otorhinolaryngol Head Neck Dis 133 (2016), S7–S11. 27256963 10.1016/j.anorl.2016.04.012

[15] Enticott J.C. , Tari S. , Koh S.M. , Dowell R.C. and O’Leary S.J. , Cochlear implant and vestibular function, Otol Nurotol 27 (2006), 824–830. 10.1097/01.mao.0000227903.47483.a6 16936568

[16] Fayad J.N. , Makarem A.O. and Linthicum F.H. , Histopathologic assessment of fibrosis and new bone formation in implanted human temporal bones using 3D reconstruction, Otol Neurotol 141 (2009), 247–252. 10.1016/j.otohns.2009.03.031 PMC2779735 19643260

[17] Fina M. , Skinner M. , Goebel J.A. , Piccirillo J.F. , Neely J.G. and Black O. , Vestibular dysfunction after cochlear implantation, Otol Neurotol 24 (2003), 234–242. 12621338 10.1097/00129492-200303000-00018

[18] Gan R.Z. , Sun Q. , Dyer R.K. , Chang K.H. and Dormer K.J. , Three-dimensional modeling of middle ear biomechanics and its application, Otol Neurotol 23 (2002), 271–280. 11981381 10.1097/00129492-200205000-00008

[19] Gundersen T. and Hogmoen K. , Holographic vibration analysis of the ossicular chain, Acta Otolaryngol 82 (1976), 16–25. 948981 10.3109/00016487609120858

[20] Gupta A. and Raj P. , Compensated vestibular dysfunction post cochlear implantation in children with sensorineural hearing loss: a prospective study, Indian J Otolaryngol Head Neck Surg 2 (2018), 200–204. 10.1007/s12070-017-1054-0 PMC6015564 29977841

[21] Huygen P.L. and Van den Broek P. , Vestibular function pre- and post-cochlear implantation, J Otolaryngol 24 (1995): 262. 8551542

[22] Inouse A. , Iwasaki S. , Ushio M. , Chihara Y. , Fujimoto C. , Egami N. and Yamasoba T. , Effect of vestibular dysfunction on the development of gross motor function in children with profound hearing loss, Audiology & neuro-otology 18 (2013), 143–151. 23392310 10.1159/000346344

[23] Jacot E. , Van Den Abbeele T. , Debre H.R. and Wiener-Vacher S.R. , Vestibular impairments pre- and post-cochlear implant in children, International journal of pediatric otorhinolaryngology 73 (2009), 209–217. 19101044 10.1016/j.ijporl.2008.10.024

[24] Jin Y. , Nakamura M. , Shinjo Y. and Kaga K. , Vestibular-evoked myogenic potentials in cochlear implant children, Acta Otolaryngol 126 (2006), 164–169. 16428194 10.1080/00016480500312562

[25] Jutila T. , Aalto H. and Hirvonen T.P. , Cochlear implantation rarely alters horizontal vestibulo-ocular reflex in motorized head impulse test, Otol Neurotol 34 (2013), 48–52. 23151779 10.1097/MAO.0b013e318277a430

[26] Kaga K. , Vestibular compensation in infants and children with congenital and acquired vestibular loss in both ears, Int Ped ORL 49 (1999), 214–224. 10.1016/s0165-5876(99)00206-2 10519701

[27] Katsiari E. , Balatsouras D.G. , Sengas J. , Riga M. , Korres G.S. and Xenelis J. , Influence of cochlear implantation on the vestibular function, Eur Arch Otorhinolaryngol 270 (2013), 489–495. 22481544 10.1007/s00405-012-1950-6

[28] Lammers M.J.W. , van der Heijden G.J.M.G. , Pourier V.E.C. and Grolman W. , Bilateral cochlear implantation in children: a systematic review and best-evidence synthesis, Laryngoscope 124 (2014), 1694–1699. 24390811 10.1002/lary.24582

[29] Li X. , Gong S.S. , The effect of 615 cochlear implantation on vestibular evoked myogenic potential in children, Laryngoscope (2020), e918–925. 10.1002/lary.28520 PMC7754474 32031698

[30] Licameli G. , Zhou G. and Kenna M.A. , Disturbance of vestibular function attribuatable to cochlear implantation in children, Laryngoscope 119 (2009), 740–745. 19205016 10.1002/lary.20121

[31] Maes L. , Kegel A.D. , van Waelvelde H. and Dhooge I. , Association between vestibular function and motor performance in hearing-impaired children, Otol Neurotol 35 (2014), e343–e347. 25275872 10.1097/MAO.0000000000000597

[32] Mattingly J.K. , Uhler K.M. and Cass S.P. , Air-bone gaps contribute to functional hearing preservation in cochlear implantation, Otol Neurotol 37 (2016), 1255–1262. 27518207 10.1097/MAO.0000000000001171

[33] Melvin T.A.N. , Della Santina C.C. , Carey J.P. and Migliaccio A.A. , The effects of cochlear implantation on vestibular function, Otol Neurotol 30 (2009), 87–94. 19108038 10.1097/mao.0b013e31818d1cba PMC2767271

[34] Merchant G.R. , Schulz K.M. , Patterson J.N. , Fitzpatrick D. and Janky K.L. , Effect of cochlear implantation on vestibular evoked myogenic potentials and wideband acoustic immittance, Ear Hear 41 (2020), 1111–1124. 32032225 10.1097/AUD.0000000000000831 PMC7392788

[35] Mondini C. , Minor works of Carlo Mondini: the anatomical section of a boy born deaf, Am J Otol 18 (1997), 288–293. 9149819

[36] Nashner L.M. , Practical biomechanics and physiology of balance, In: Jacobson P, Newman CW, Kartush JM, eds. Handbook of balance functions testing. 1 st ed. Chicago: Singular (1993), 261–279.

[37] Nassif N. , Balzanelli C. and Redaelli de Zinis L.O. , Preliminary results of video Head Impulse Testing (vHIT) in children with cochlear implants, International journal of pediatric otorhinolaryngology 88 (2016), 30–33. 27497382 10.1016/j.ijporl.2016.06.034

[38] Nomura Y. , Morphological aspects of inner ear disease, Springer Japan (2014).

[39] Parkes W.J. , Gnanasegaram J.J. , Cushing S.L. , Mcknight C.L. , Papsin B.C. and Gordon K.A. , Vestibular evoked myogenic potential testing as an objective measure of vestibular stimulation with cochlear implants, Laryngoscope 127 (2017), E75–81. 27291637 10.1002/lary.26037

[40] Raveh E. , Attias J. , Nageris B. , Kornreich L. and Ulanovski D. , Pattern of hearing loss following cochlear implantation,72, Eur Arch Otorhinolaryngol 2 (2015), 2261–2266. 10.1007/s00405-014-3184-2 25012703

[41] Rine R.M. and Christy J.B. , Physical therapy management of children with vestibular dysfunction, In: Herdman SJ, Clendaniel RA, eds. Vestibular Rehabilitation. 4th ed. Philadelphia: F.A. Davis Company (2014), 457.

[42] Robard L. , Hitier M. , Lebas C. and Moreau S. , Vestibular function and cochlear implant, Eur Arch Otorhinolaryngol 272 (2015), 523–530. 24737055 10.1007/s00405-014-3040-4

[43] Roland P.S. , Wright C.G. and Isaacson B. , Cochlear implant electrodes insertion: the round window revisited, Laryngoscope 117 (2007), 1397–1402. 17585282 10.1097/MLG.0b013e318064e891

[44] Sheykholeslami K. , Schmerber S. , Kermany M.H. and Kaga K. , Vestibular-evoked myogenic potentials in three patients with large vestibular aqueduct, Hearing Research 190 (2004), 161–168. 15051138 10.1016/S0378-5955(04)00018-8

[45] Tamir S. , Ferrary E. , Borel S. , Sterkers O. and Grayeli A.B. , Hearing preservation after cochlear implantation using deeply insertion flex atraumatic electrode arrays, Audiol Neurootol 17 (2012), 331–337. 22813984 10.1159/000339894

[46] Taylor R.L. , Bradshaw A.P. , Magnussen J.S. , Gibson W.P.R. , Halmagyi G.M. and Welgampola M.S. , Augmented ocular vestibular evoked myogenic potentials to air-conducted sound in large vestibular aqueduct syndrome, Ear and Hearing 33 (2012), 768–771. 22836238 10.1097/AUD.0b013e31825ce613

[47] Thierry B. , Blanchard M. , Leboulanger N. , Parodi M. , Wiener-Vacher S.R. , Garabedian E.N. and Loundon N. , Cochlear implantation and vestibular function in children, International Journal of Pediatric Otorhinolaryngology 79 (2015), 101–104. 25500550 10.1016/j.ijporl.2014.11.002

[48] Tien H.C. and Linthicum F.H. Jr , Histopathologic changes in the vestibule after cochlear implantation, Otolaryngol Head Neck Surg 127 (2002), 260–264. 12402002 10.1067/mhn.2002.128555

[49] Tomaski S.M. , Zalzal G.H. and Saal H.M. , Airway obstruction in the Pierre Robin sequence, Laryngoscope 105 (1995), 111–114. 8544588 10.1288/00005537-199502000-00001

[50] Tsukada K. , Moteki H. , Fukuoka H. , Iwasaki S. and Usami S.I. , Effects of EAS cochlear implantation surgery on vestibular function, Otolaryngol 133 (2013), 1128–1132. 10.3109/00016489.2013.824110 PMC3809927 24007563

[51] Uzun H. , Curthoys I.S. and Jones A.S. , A new approach to visualizing the membranous structures of the inner ear- high resolution X-ray micro-tomography, Acta Otolaryngol 127 (2007), 568–573. 17503224 10.1080/00016480600951509

[52] Weber K.P. , MacDougall H.G. , Halmagyi G.M. and Curthoys I.S. , Impulse testing of semicircular-canal function using video-oculograpy, Annals of the New York Academy of Sciences 1164 (2009), 486–491. 19645955 10.1111/j.1749-6632.2008.03730.x

[53] Xu X.D. , Zhang X.T. , Zhang Q. , Hu J. , Chen Y.F. and Xu M. , Ocular and cervical vestibular-evoked myogenic potentials in children with cochlear implant, Clin Neurophysiol 126 (2015), 1624–1631. 25511635 10.1016/j.clinph.2014.10.216

[54] Yong M. , Young E. , Lea J. , Foggin H. , Zaia E. , Kozak F.K. and Westerberg B.D. , Subjective and objective vestibular changes that occur following paediatric cochlear implantation: systematic review and meta-analysis, J Otolaryngol Head Neck Surg 48 (2019), 22. 31118089 10.1186/s40463-019-0341-z PMC6530180

[55] Zalewski C.K. , Chien W.W. , King K.A. , Muskett J.A. , Baron R.E. , Butman J.A. , Griffith A.J. and Brewer C.C. , Vestibular dysfunction in patients with enlarged vestibular aqueduct, Otolaryngol Head Neck Surg 153 (2015), 257–262. 25968061 10.1177/0194599815585098 PMC8010515

[56] Zellhuber S. , Mahringer A. and Rambold H.A. , Relation of video-head-impulse test and caloric irrigation: a study on the recovery in unilateral vestibular neuritis, Eur Arch Otorhinolaryngol 271 (2014), 2375–2383. 24096811 10.1007/s00405-013-2723-6

[57] Zhou Y.J. , Wu Y.Z. , Cong N. , Yu J. , Gu J. , Wang J. and Chi F.L. , Contrasting results of tests of peripheral vestibular function in patients with bilateral large vestibular aqueduct syndrome, Clin Neurophysiol 128 (2017), 1513–1518. 28667933 10.1016/j.clinph.2017.05.016

